# Enantioselective hyperporous molecularly imprinted thin film polymers†

**DOI:** 10.1039/c9ra07425b

**Published:** 2019-10-18

**Authors:** Sofia M. E. Nilsson, Subramanian Suriyanarayanan, Subban Kathiravan, Jari Yli-Kauhaluoma, Tapio Kotiaho, Ian A. Nicholls

**Affiliations:** Drug Research Program, Division of Pharmaceutical Chemistry and Technology, Faculty of Pharmacy, University of Helsinki P. O. Box 56 (Viikinkaari 5 E) FI-00014 Finland; Bioorganic & Biophysical Chemistry Laboratory, Linnaeus University Centre for Biomaterials Chemistry, Linnaeus University SE-39182 Kalmar Sweden ian.nicholls@lnu.se; Department of Chemistry, Faculty of Science, University of Helsinki P. O. Box 55 (A. I. Virtasen aukio 1) FI-00014 Finland

## Abstract

Significant enantioselective recognition has been achieved through the introduction of long range ordered and highly interconnected 300 nm diameter pores in molecularly imprinted polymer matrices.

## Graphical abstract

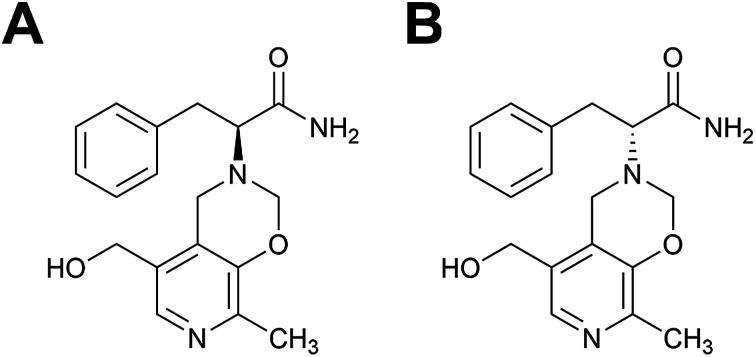

For applications such as heterogenous catalysis, separation and surface-based sensing, enhancing numbers of recognition sites and diffusion to and from these sites are factors critical for improving material performance.^1–6^ Examples include the incorporation of reactive or ligand-selective functionality in porous inorganic matrices, *e.g.* zeolites,^7,8^ super porous silica,^9,10^ anodized alumina,^11,12^ and synthetic polymers by chemical functionalization/grafting.^13,14^

Ligand-selective recognition sites can be generated in a polymer matrix by molecular imprinting.^15,16^ The recognition properties of molecularly imprinted materials have led to them being used in a wide range of applications.^17,18^ With our long-term objective to develop lab-on-a-chip mini-reactors incorporating catalytic molecularly imprinted polymers (MIPs), where the issues of active site density and mass transfer are critical, we endeavored to explore the possibility of enhancing MIP catalytic functionality through combining the imprinting technique with the use of a sacrificial scaffold. MIPs capable of facilitating chemical reactions have been prepared using a strategy akin to that used in the development of catalytic antibodies,^19,20^ whereby a transition state analogue (TSA) is used as template during the MIP synthesis. MIPs have been developed for a wide range of chemical transformations,^21^ such as hydrolysis,^22^ oxidation,^23^ [2 + 2] photodimerization,^24^ cycloaddition^25^ and asymmetric hydrogenation.^26^ For many applications, including catalysis, numbers of high affinity sites and mass transfer are issues limiting performance.^27,28^ The synthesis of imprinted polymers with structural features that facilitate ligand access to recognition sites has been explored using polymer synthesized with sacrificial scaffolds, *e.g.* silica,^29^ anodized alumina,^30^ micelles^31^ and monodisperse (latex) polystyrene (PS) beads.^32,33^ These strategies have been successfully used to enhance the performance of surface-based sensing techniques, *e.g.* quartz crystal microbalance (QCM),^32^ and electrochemical methods.^12^

For this study we selected a previously described MIP capable of catalyzing a transamination reaction,^34^ the physiologically important synthesis of an α-amino acid from the corresponding α-ketocarboxylic acid, in this case the reaction of phenylpyruvic acid and pyridoxamine to yield phenylalanine and pyridoxal^34^ (Scheme S1†). The enantiomers of a TSA for this reaction were used as templates in the MIP synthesis, Fig. 1. Features of this polymer system include its enantioselectivity, whereby the choice of template chirality gave the resultant methacrylic acid (MAA)–ethylene glycol dimethacrylate (EGDMA) copolymer with the corresponding enantioselectivity, and catalytic functionality in aqueous media.

**Fig. 1:**
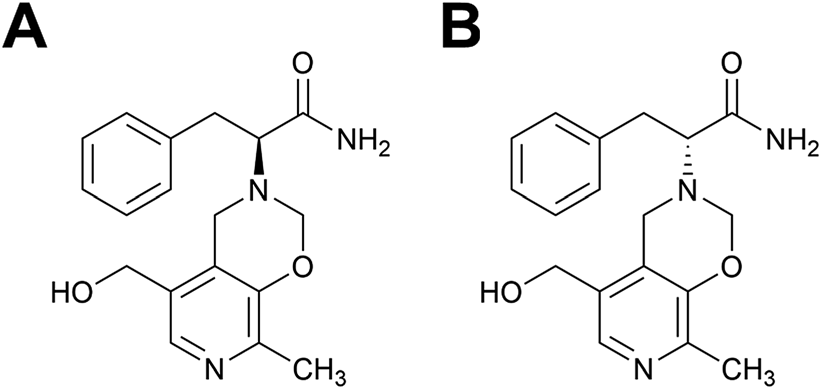
Structures of the (A) l- and (B) d-transition state analogues for the reaction of phenylpyruvic acid and pyridoxamine to yield phenylalanine and pyridoxal, as first proposed by Winkler *et al.*^35^

The regular highly porous structures previously prepared using sacrificial self-assembled monodisperse polystyrene beads were perceived to offer good potential for enhancing diffusion and thus template binding site accessibility.^32,33^ However, the solubility of the polystyrene beads in the porogen previously used in the MIP synthesis, chloroform, necessitated an alternative solvent, that while not dissolving the polystyrene beads was still capable of solubilizing the polymerization reaction mixture components. The screening of solvents with dielectric constants below that of chloroform (4.8 at 293 K), led to the identification of *n*-heptane (1.9 at 293 K) as a solvent that could solubilize polymerization reaction components without increasing competition for electrostatic interactions between template and monomer and without dissolving the polystyrene beads, which are normally removed by dissolving in toluene (2.4 at 293 K).

To ascertain the impact of the sacrificial scaffold on imprinted polymer recognition characteristics in a QCM microfluidics device, a series of polymer films was prepared on SiO_2_@Au-coated quartz crystal resonators (≈0.12 cm^2^ in area), Fig. 2. The investigated polymer systems were either prepared in the presence of *n*-heptane as solvent or without any solvent, with and without the presence of self-assembled monodisperse 300 nm PS beads, and either in the presence, or absence of the l-TSA template, Tables 1 and S1.† The self-assembled beads showed long-range order, as seen in scanning electron microscopy (SEM) studies, Fig. 3D and S1.† Interestingly, our initial studies to establish the chemistry used microscope slides (≈15 cm^2^ in area), highlighting the potential for producing these materials in larger formats.

**Fig. 2:**
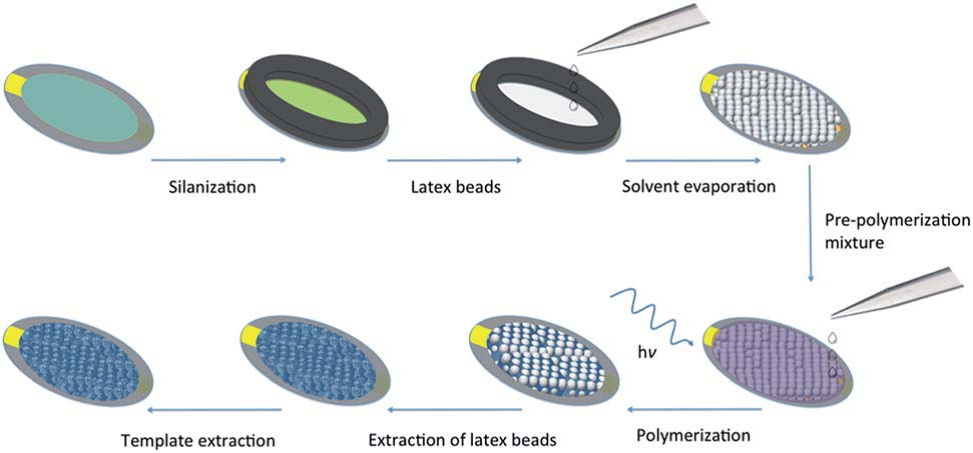
Schematic representation of the strategy used for preparing hyperporous molecularly imprinted polymer films using sacrificial polystyrene (latex) beads on QCM resonators.

**Table 1:** Poly(MAA-*co*-EGDMA) systems synthesized and analyzed in this study

Polymer system	Template	Sacrificial scaffold	Solvent
P1	—	PS beads	*n*-Heptane
P2	—	—	*n*-Heptane
P3	—	PS beads	—
P4	l-TSA	PS beads	*n*-Heptane
P5	l-TSA	—	*n*-Heptane
P6	l-TSA	PS beads	—
P7	d-TSA	PS beads	*n*-Heptane
P8	—	—	—
P9	l-TSA	—	—

**Fig. 3:**
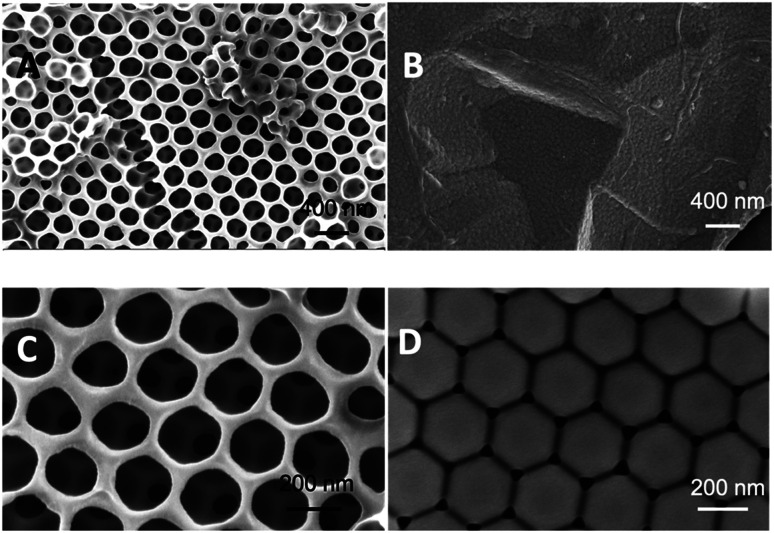
SEM images of l-TSA imprinted polymer systems prepared in the presence and absence of polystyrene beads and *n*-heptane solvent, respectively. (A) P4, (B) P5 and (C) P6. (D) is the SEM image of self-assembled 300 nm diameter polystyrene beads drop coated on SiO_2_@Au-coated quartz crystal resonators.

SEM studies of the polymers showed the presence of long-range ordered interconnected networks of pores (Fig. 3A and C) strongly reflecting the layers of sacrificial self-assembled latex beads (Fig. S2 and S3†). Importantly, this indicates that the polymer bead assemblies remain largely intact throughout the polymerization process. Polymer film (P5) prepared in the absence of beads was devoid of macroporous structure (Fig. 3B). Upon comparison of the SEM images of P4, synthesized using *n*-heptane and sacrificial bead scaffold, and P6, using no porogen, the later produced a somewhat more regularly structured polymer network (S2B *vs.* S3B†). This was concluded to arise from the more intimate interaction of monomers with the polystyrene beads. Studies (data not presented) using 100 nm diameter beads produced polymer films though with significantly less ordered pores.

Importantly, Fourier-transform infrared spectroscopy (FTIR) for the polymer films prepared in the absence and presence of sacrificial bead layers display similar spectral properties (Fig. S4†), in particular bands *ν*C=O (1710 cm^−1^), *δ*(C–H) (1455 cm^−1^), *ν*C–O (1145 and 1250 cm^−1^) and *ρ*C–H (750 and 900 cm^−1^) arising from vibrational modes. This data also indicate that the presence and removal of the polymer beads has no apparent influence on the chemical composition of the resultant MIP films. Support for the effective removal of the beads is seen, where the strong bands at 696, 752, 1216, 1449, 1492, 1602, 2922 and 3025 cm^−1^, as well as overtones around 1800–2000 cm^−1^, arising from the polystyrene beads effectively disappear after treatment with toluene (Fig. S4A†). The thickness of the films, 26–100 μm, was controlled by regulating the amounts of the polymerization mixture components used, including where relevant the polystyrene beads, and calculated as described in the ESI (S9).†

The recognition properties of the polymer-coated resonators were initially explored by flow injection analysis (FIA) using a QCM, and a 1 : 1 solution of methanol–aqueous sodium acetate (0.1 M, pH 7.0) as mobile phase, by determining the sensitivities of the polymer films to the templates; the l-TSA template and its enantiomer, d-TSA, Fig. 3 and S5.† Comparison of the polymer films prepared in the presence of the sacrificial bead scaffold and either in the absence or presence of the l-TSA imprinted in *n*-heptane, P1 and P4, respectively, showed a clear selectivity for the l-TSA over its enantiomer in the case of P4. No significant enantioselectivity was observed in the case of the non-imprinted P1, with small observed differences in calculated sensitivities being within experimental error for these studies, ±0.04 Hz mM^−1^. The importance of the sacrificial bead scaffold, and even the presence of porogen, on the capacity and enantioselectivity of the polymers was evident upon comparison with the l-TSA imprinted and non-imprinted polymers prepared in the absence of the bead scaffold, P2 and P5, or the porogen, P3 and P6. In these cases, no significant template enantioselectivity was observed, which highlighted the importance of the collective impact of the porogen and sacrificial bead scaffold-induced interconnected pores on access to template-selective binding sites. To confirm this hypothesis, a polymer film was prepared using the l-TSA as template with *n*-heptane as porogen and the sacrificial bead scaffold, P7. This polymer demonstrated a selectivity for the d-TSA over l-TSA – the opposite to that seen for P4, thus substantiating the impact of the combination of beads and porogen on template selectivity. Polymer films prepared in the absence of both porogen and sacrificial beads (zero polymers P8 and P9) demonstrated low levels of binding and sensitivity for the TSAs compared to system P4, reflecting the anticipated lack of accessible sites.

We then proceeded to investigate the broader selectivity of the polymer films towards the reactants and products for this transamination reaction (Scheme S1†). The TSAs demonstrated superior binding in all cases. This was attributed to their greater number of functional groups and presence of two aromatic rings, which are able to contribute to both the TSA-selective and non-specific binding through a combination of electrostatic and hydrophobic binding interactions, Fig. 4 and S5.† In all cases, the sensitivities of the other analytes (Scheme S1†) were lower, reflecting less selective binding. This data is in general accordance with the selectivity profile of the previously reported bulk polymers synthesized using chloroform.^34^ It was interesting to note that the phenylpyruvic acid was better recognized by the hyperporous TSA-imprinted polymers, P4 and P7, than on the other polymers. This suggests that it accesses the binding domains derived from the TSA's aromatic rings, a process favorable in the aqueous rebinding media.

**Fig. 4:**
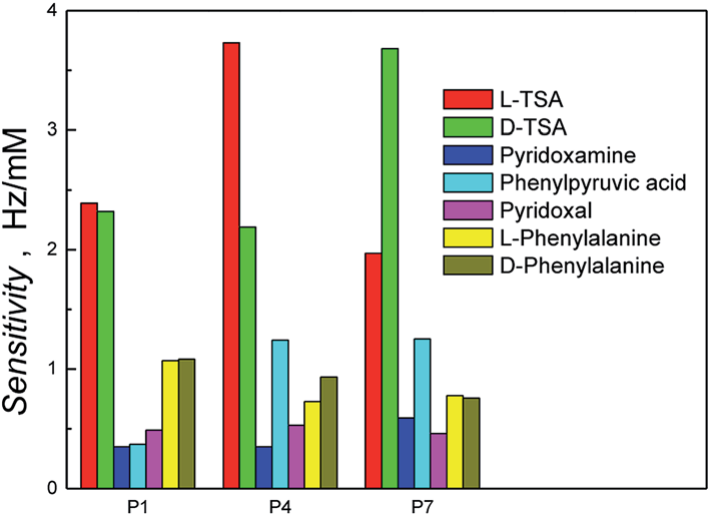
Histogram of sensitivity of the polymer systems (P1, P4 and P7 from Table 1) obtained from the slope of the flow injection analysis (FIA) calibration plots of various analytes on different polymer systems.

In summary, long-range, highly-porous structural features in molecularly imprinted polymer thin films can be used to enhance access to template binding sites, as exemplified through the significant enantioselectivity revealed upon combining the use of these sacrificial scaffolds in conjunction with molecular imprinting using a suitable porogen. These hyperporous morphologies can be easily obtained using sacrificial self-assembled monodisperse polystyrene bead-based scaffolds. The resultant enhanced access to recognition sites should prove useful in applications requiring facile mass transfer such as in surface-based sensing techniques and for heterogenous catalysis. Furthermore, this technique is scalable, and readily applicable to substrates of 15 cm^2^ in area. The enhancement of enantioselectivity obtained using these hierarchical material architectures highlights the importance of site accessibility for analyte binding to molecularly imprinted materials. These hyperporous polymer films demonstrated no significant change in their recognition characteristics after 72 h of use in the QCM instrument. Moreover, this fabrication strategy should prove useful for enhancing MIP performance, in particular for their use in applications where binding site numbers and access are otherwise limiting factors.

## Conflicts of interest

The authors declare no conflicts of interest.

## Supplementary Material

RA-009-C9RA07425B-s001
