# Supplementary material for: Enantioselective hyperporous molecularly imprinted thin film polymers
Source: RSC Adv. 2019 Oct 18;9(58):33653–6. doi: 10.1039/c9ra07425b (PMC9073540; doi:10.1039/c9ra07425b)
Supplement: RA-009-C9RA07425B-s001 [file RA-009-C9RA07425B-s001.pdf]

Electronic Supplementary Material (ESI) for RSC Advances.
This journal is © The Royal Society of Chemistry 2019

Electronic Supplementary Information

Enantioselective hyperporous molecularly imprinted thin film polymers

Sofia M. E. Nilsson, [a] *Subramanian Suriyanarayanan,* [b] *Subban Kathiravan,* [b] *Jari Yli-Kauhaluoma,* [a] *Tapio Kotiaho* [a, c] *and Ian A. Nicholls* [b] *

[a.] Drug Research Program, Division of Pharmaceutical Chemistry and Technology, Faculty of Pharmacy, P.O. Box 56 (Viikinkaari 5 E), FI-00014 University of Helsinki, Finland

[b.] Bioorganic & Biophysical Chemistry Laboratory, Linnaeus University Centre for Biomaterials Chemistry, Linnaeus University, SE-391 82 Kalmar, Sweden

[c.] Department of Chemistry, Faculty of Science, P.O. Box 55 (A.I. Virtasen aukio 1), FI-00014 University of Helsinki, Finland

*Corresponding author e-mail: ian.nicholls@lnu.se

Table of Contents

1. Chemicals....S3
   1.1 Chemicals used for synthesizing the transition state analogues S3
   1.2 Chemicals used for cleaning quartz crystal microbalance chips, borosilicate- and silica substrates....S3
   1.3 Chemicals used for the QCM experiments S3
2. Synthesis procedures S4
   2.1 Synthesis procedures for preparing the transition state analogues S4
   2.1.1 Characterization of the synthesized transition state analogues S5
   2.2 Procedures for preparing the QCM chips S7
   2.3 Procedure for preparing the borosilicate and silicon substrates S8
3. QCM experiments S10
   3.1 QCM experiments set-up....S10
   3.2 Analyte solutions for the QCM experiments S10
4. Scanning electron microscopy S10
5. Fourier-transform infrared spectroscopy....S11
6. References S11

Scheme S1	S12
Figure S1	S13
Figure S2	S14
Figure S3	S15
Figure S4	S16
Figure S5	S17
Table S1	S18
Table S2	S19

1. Chemicals

1.1 Chemicals used for synthesizing the transition state analogues

L- and D-phenylalaninamide, pyridoxal hydrochloride (≥99%), methanol and acetonitrile (HPLC grade) and *d*-chloroform (>99.8 atom % D) and d_6-DMSO (>99.9 atom % D) were purchased from Chemtronica or Sigma-Aldrich. The L-TSA and D-TSA were both prepared according to a previously reported procedure[1]. ^{1}H and ^{13}C nuclear magnetic resonance (NMR) spectroscopy, and liquid chromatography mass spectrometry (LC-MS) were used to characterize all isolated compounds. The characterization data correspond with the previously reported one[1]. NMR spectra were recorded on a Varian 500 MHz instrument. All ^{1}H NMR experiments are reported in the unit parts per million (ppm), and were measured relative to the signal for residual solvent, $CDCl_3$ (7.26 ppm). All ^{13}C NMR spectra are reported in ppm relative to solvent, DMSO-d_6 (39.52 ppm), unless otherwise stated, and all were obtained with ^{1}H decoupling. The coupling constants are reported in Hertz (Hz).

1.2 Chemicals used for cleaning quartz crystal microbalance chips, borosilicate- and silica substrates

The following solvents were used: Toluene (laboratory reagent grade, Sigma-Aldrich), ethanol (99.5%, analytical grade, Solveco AB for cleaning the quartz crystal microbalance (QCM) chips, silicon and borosilicate substrates, and 80% reagent grade, Sigma-Aldrich for cleaning the Fourier-transform infrared spectroscopy instrument), methanol (reagent Ph. Eur. grade, Carlo Erba, DASH group), acetone (technical grade, VWR), tetrahydrofuran (Chromasolv®–grade, Sigma-Aldrich), acetonitrile (HPLC grade, Carlo Erba reagents, DASH group), *n*-heptane (laboratory reagent grade, Sigma-Aldrich) and deionized water (purified with a Milli-Q system from Millipore). Only solvents which had been dried with molecular sieves (4 Å, 3.2 mm pellets, Sigma-Aldrich) were used (except for methanol and ethanol which were used un-dried). Hydrogen peroxide (30% w/w in water, Sigma-Aldrich), sulfuric acid (95-98%, Sigma-Aldrich), hydrogen chloride solution (37%, fuming, Merck) and ammonia solution (25%, Merck) were used to clean the QCM chips, and/or the borosilicate substrates and the silica substrates. 3-(Trimethoxysilyl)propyl methacrylate (98%, Sigma-Aldrich) and triethylamine (98%, Alfa Aesar) were used for the silanization of the QCM chips, borosilicate- and silica substrates. Methacrylic acid (MAA, 99%) and ethyl glycol dimethylacrylate (EGDMA, 98%) were obtained from Sigma-Aldrich and distilled before use, and azobisisobutyronitrile (AIBN, 98%) was bought from Sigma-Aldrich and re-crystallized from methanol before use. Polystyrene (latex) beads with the mean diameter 0.3 µm (range: 0.30-0.33 µm, standard deviation 0.03-0.05 µm) in a 10% w/w water suspension, were purchased from Sigma-Aldrich.

1.3 Chemicals used for the QCM experiments

Pyridoxamine dihydrochloride (≥98%, **6**), phenylpyruvic acid (98%, **7**), pyridoxal hydrochloride (≥99%, **2**), L-phenylalanine (≥99.0%, **L-11**) and D-phenylalanine (>98%, **D-11**) were all purchased

from Sigma-Aldrich. Sodium acetate (NaOAc, 99.3%) was obtained from VWR. The L-TSA and D-TSA were synthesized in-house as described in section S2, and their identity has been confirmed with NMR, MS and melting point analysis (see section S2.1.1 for details). A 0.1 M solution of NaOAc in H_2O (pH 7), a 5 mM solution of NaOH in H_2O and a 0.1 M solution of NaOH in H_2O were also prepared in-house.

2. Synthesis procedures

2.1 Synthesis procedures for preparing the transition state analogues

(*S*)-2-[[[3-Hydroxy-5-(hydroxymethyl)-2-methylpyridin-4-yl]methyl]amino]-3-phenylpropanamide (3)

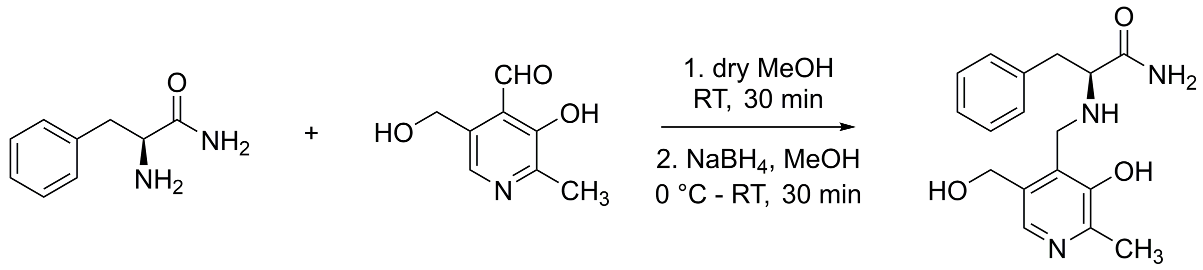

1 **2** **3**

To a solution of L-phenylalaninamide **1** (1.64 g, 9.9 mmol) in methanol (110 mL) was added pyridoxal hydrochloride **2** (2.04 g, 9.9 mmol) and the resulting solution was stirred at room temperature for 30 min. After this, the yellow solution was cooled on ice bath. Sodium borohydride (0.38 g, 10.0 mmol) was added in portions (Caution! Effervescence was noticed) to the yellow solution. The deep yellow solution was stirred for another 30 min at room temperature. The solvent was evaporated under reduced pressure. The resulting yellow residue was subjected to flash chromatography on silica gel using acetonitrile:H_2O (80:20, v/v) as eluent to give the amine **3** as yellow solid in 75% yield. The characterization data are in accordance with those previously reported.[1]

(*R*)-2-[[[3-Hydroxy-5-(hydroxymethyl)-2-methylpyridin-4-yl]methyl]amino]-3-phenylpropanamide was synthesized as described above, using D-phenylalaninamide as starting material instead of L-phenylalaninamide.

(*S*)-2-[5-Hydroxymethyl-8-methyl-2*H*-pyrido[4,3-*e*][1,3]-oxazin-3(4*H*)-yl)]3-phenylpropanamide (L-TSA) (4)

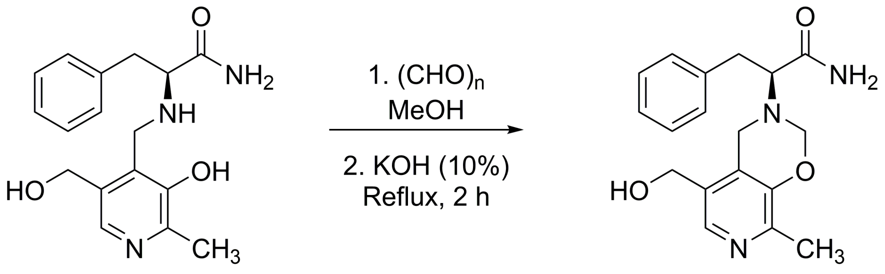

3 **4**

To a solution of paraformaldehyde (0.84 g, 28 mmol) in methanol (120 mL), KOH (10 mol%) and amine **3** (2.39 g, 7.6 mmol) were added under nitrogen atmosphere and the resulting solution was refluxed for 2 h. The solution was cooled, concentrated and purified by flash chromatography using acetonitrile:H_2O (80:20, v/v) as eluent. The desired product (R_f 0.70) was extracted using dichloromethane and brine to give L-TSA as pale yellow solid in 56% yield.

The D-TSA was prepared in 51% yield, following the procedure for L-TSA[1].

2.1.1 Characterization of the synthesized transition state analogues

(*S*)-2-[5-Hydroxymethyl-8-methyl-2*H*-pyrido[4,3-*e*][1,3]-oxazin-3(4*H*)-yl)]3-phenylpropanamide (L-TSA) (4)

Color: Pale yellow solid; Mp: 93-97 °C; ^{1}H NMR (500 MHz, Chloroform-*d*) δ 7.90 (s, 1H), 7.23 (d, J = 7.0 Hz, 2H), 7.21–7.15 (m, 3H), 6.36 (s, 1H), 5.61 (s, 1H), 5.05 (d, J = 10.1 Hz, 1H), 4.87 (d, J = 10.3 Hz, 1H), 4.50 (s, 2H), 4.28 (d, J = 17.9 Hz, 1H), 4.14 (d, J = 18.0 Hz, 1H), 3.74 (t, J = 6.9 Hz, 1H), 3.23 (dd, J = 13.9, 7.0 Hz, 1H), 3.10 (d, J = 7.4 Hz, 1H), 2.37 (s, 3H); ^{13}C NMR (126 MHz, DMSO-d_6) δ 172.92, 148.94, 145.56, 138.89, 138.86, 132.17, 129.53, 128.50, 127.78, 126.50, 81.08, 65.80, 58.84, 44.82, 35.98, 19.04; LC-MS Calculated $[M+H]^+$ 328.2 found 328.1.

(*R*)-2-[5-Hydroxymethyl-8-methyl-2*H*-pyrido[4,3-*e*][1,3]-oxazin-3(4*H*)-yl)]3-phenylpropanamide (D-TSA) (5)

Color: Pale yellow solid; Mp: 93-97 °C; ^{1}H NMR (500 MHz, Chloroform-*d*) δ 7.88 (s, 1H), 7.25–7.15 (m, 5H), 6.35 (s, 1H), 5.62 (s, 1H), 4.95 (dd, J = 91.3, 10.1 Hz, 2H), 4.50 (s, 2H), 4.31–4.09 (m, 2H), 3.73 (t, J = 7.1 Hz, 1H), 3.22 (dd, J = 14.1, 7.1 Hz, 1H), 3.07 (dd, J = 14.0, 6.9 Hz, 1H), 2.35 (s, 3H); ^{13}C NMR (126 MHz, DMSO-d_6) δ 172.93, 148.96, 145.56, 138.90, 138.85, 132.19, 129.54, 128.51, 127.82, 126.51, 81.10, 65.82, 58.84, 44.83, 36.00, 19.04; LC-MS Calculated $[M+H]^+$ 328.2 found 328.1.

^{1}H NMR of L-TSA (4)

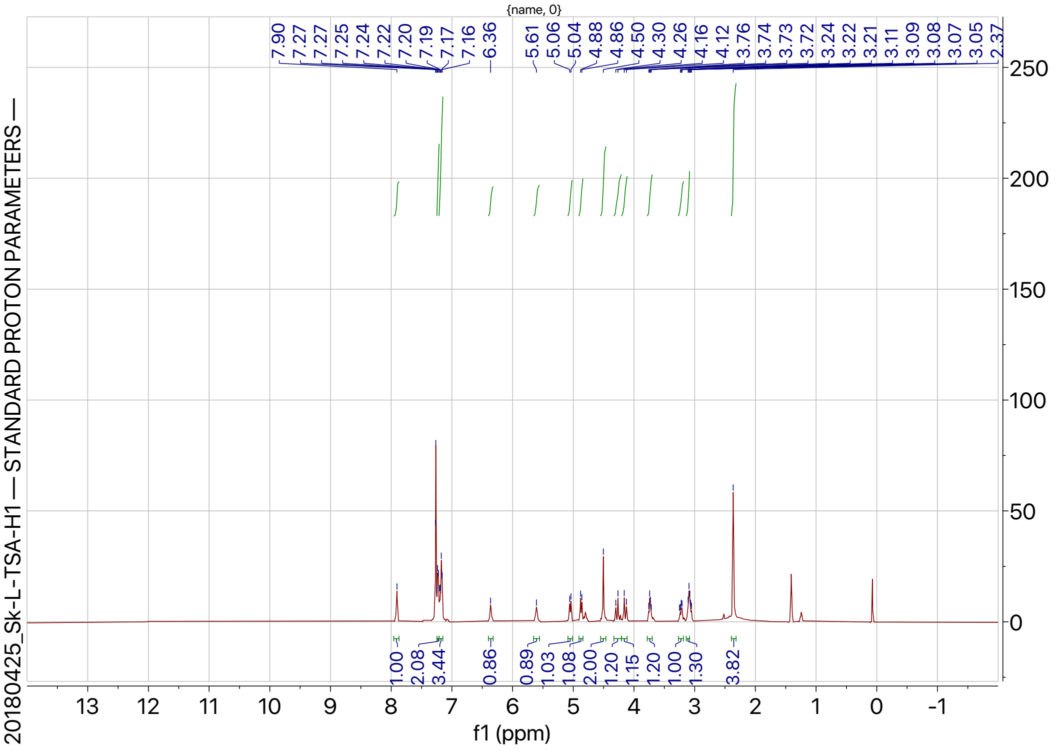

^{13}C NMR of L-TSA (4)

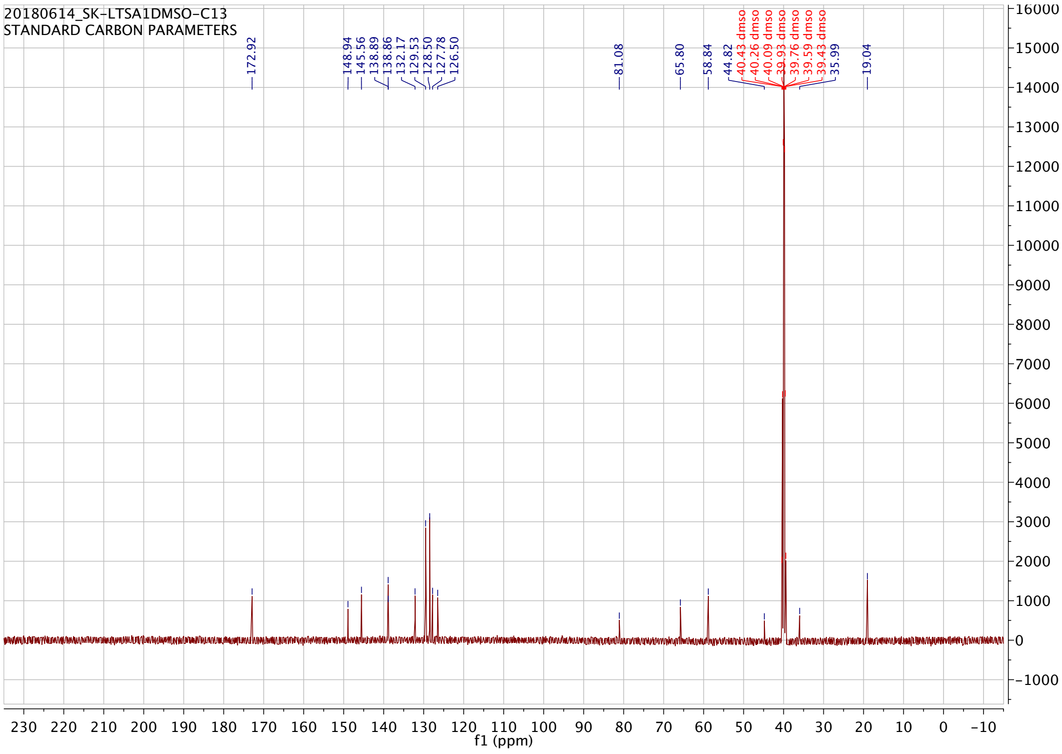

^{1}H NMR of D-TSA (5)

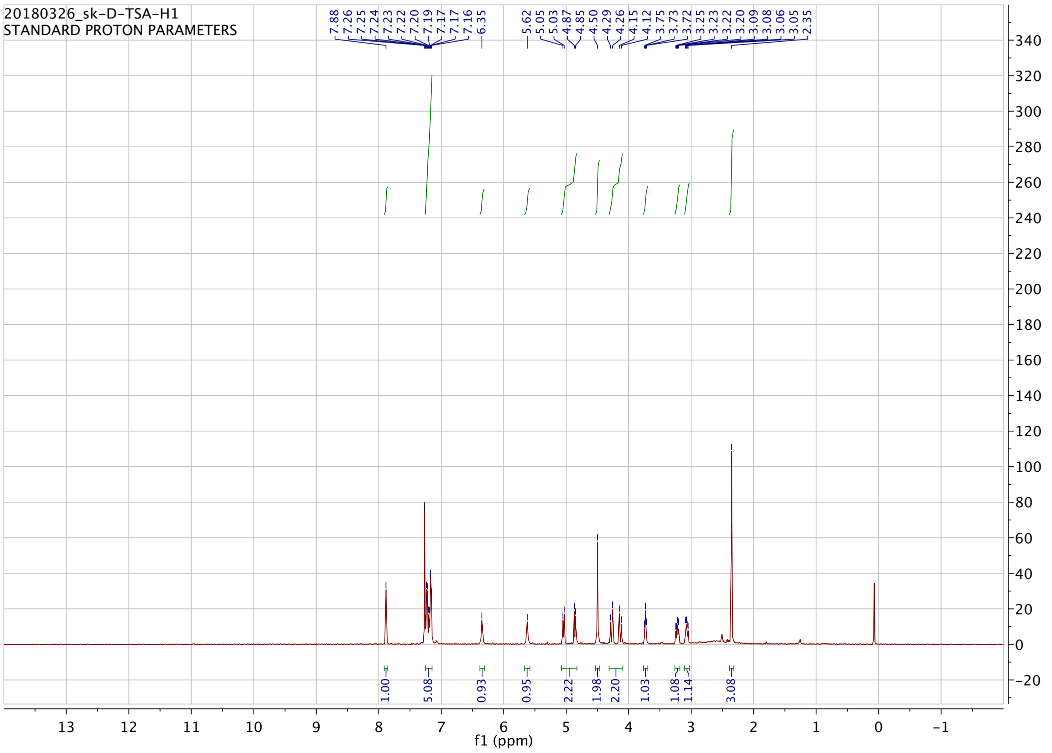

^{13}C NMR of D-TSA (5)

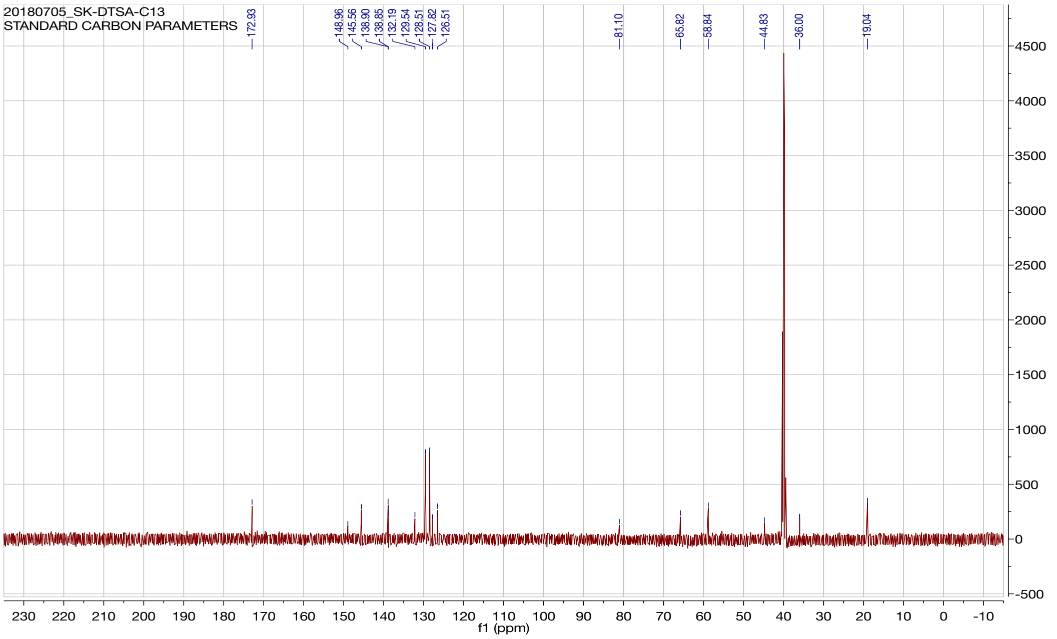

2.2 Procedures for preparing the QCM chips

Silicon dioxide (SiO_2) coated Au-quartz QCM chips (obtained from Attana AB) were sonicated for 10 min in 99.5% ethanol (1 mL), and subsequently in dry acetone (1 mL). Next, the QCM chips

were cleaned with "Piranha" solution (H_2SO_4:H_2O_2 3:1) for 1 min. (*Caution! "Piranha" solution must be handled with extreme care since it is a hazardous oxidizing agent and reacts violently with most organic materials!*) The QCM chips were sonicated for 10 min in a 0.1 M solution of NaOH in H_2O (1 mL), rinsed for 10 min in the following solvents, respectively: deionized water (1 mL), dry acetone (1 mL), dry tetrahydrofuran (1 mL), and dry toluene (1 mL).

Next, the QCM chips were immersed for 16 h in a freshly prepared solution containing 3-(trimethoxysilyl)propyl methacrylate (14.4 µL), triethylamine (1.44 µL), and dry toluene (720 µL). To clean away excessive silane, the QCM chips were rinsed with a series of 1 mL volumes of solvents for 10 min each (dry toluene, dry tetrahydrofuran, dry acetone) and were subsequently dried with nitrogen gas. An O-ring (ID 7 mm), corresponding to the diameter of the silanized surface of the QCM chip, was positioned to fence the area for silanization on the surface of the QCM chip. For the polymer systems with polystyrene, polystyrene suspension (30 µL; 0.3 µm bead diameter, 10% w/w aqueous suspension) was deposited by drop coating on the silanized area of the QCM chip (i.e. within the O-ring). After that, the drop-coated surfaces were placed in a desiccator to evaporate the solvent, leaving dry polystyrene beads on the silanized surfaces.

Prepolymerization mixtures were prepared with MAA, EGDMA and AIBN as given in Table S1. Briefly, <u>L-TSA (1.30 mg, 3.97 µmol),</u> MAA (4.04 µL, 47.6 µmol) and EGDMA (41.6 µL, 0.221 mmol) were thoroughly mixed in <u>*n*-heptane</u> (<u>74 µL)</u> [The components not included in all systems are underlined]. Then, AIBN (1.30 mg, 7.92 µmol) was added to this mixture and purged with N_2 for 10 min. N_2-purged pre-polymerization mixture (1 µL) was deposited on the polystyrene beads coated silanized QCM chip, and a cover glass was placed on top of the pre-polymerization mixture. Immediately, the QCM chip with the pre-polymerization mixture was placed under UV irradiation (50 W UV lamp, 365 nm from Labino AB) for 2 h. After that, the cover glass was removed, and the chips were rinsed for 10 min in dry toluene (1 mL), a 5 mM solution of NaOH in H_2O (1 mL), and deionized water (1 mL).

2.3 Procedure for preparing the borosilicate and silicon substrates

Borosilicate glass and silicon substrates (Sigma-Aldrich) were prepared for deposition of polymer samples for Fourier-transform infrared spectroscopy (FTIR) and scanning electron microscopy (SEM) studies, respectively, according to the following protocol. Borosilicate glass slides and silicon wafers were put in a beaker, and immersed in the following solvents:

15 mL 30% H_2O_2 + 15 mL HCl (37%, fuming) + 90 mL deionized water, 80 °C, 5 min
20 mL a 25% solution of NH_3 in H_2O + 100 mL deionized water, 80 °C, 5 min
20 mL 30% H_2O_2 + 100 mL deionized water, 80 °C, 5 min
120 mL deionized water, room temperature (RT), 5 min
120 mL deionized water, RT, 5 min
120 mL dry acetone, RT, 5 min
120 mL dry acetone, RT, 5 min

120 mL dry tetrahydrofuran, RT, 5 min
120 mL dry tetrahydrofuran, RT, 5 min
120 mL dry toluene, RT, 5 min
120 mL dry toluene, RT, 5 min

After the washing steps described above, the substrates were silanized as described above, in section S2.2. Subsequently, the systems containing polystyrene were drop-coated with polystyrene suspension (note: here two layers, i.e. 2×30 µL, of polystyrene was sequentially deposited (the second layer was deposited after the first layer had dried)), and pre-polymerization mixture was deposited on them, as described in section S2.2. The borosilicate and silicon substrates were additionally washed as described in section S2.2, with the exception that the borosilicate substrates which were drop coated with polystyrene prior to deposition of pre-polymerization mixture were washed in 99.5% ethanol, subjected to FTIR analysis, and subsequently washed in the solvents listed for washing the QCM chips after polymerization (section S2.2) before recording another FTIR spectrum. The borosilicate substrates prepared without any polystyrene and the silicon substrates were only washed in the solvents described (section S2.2) for washing the QCM chips after polymerization.

The thickness of the polymer films was estimated using the following approach:

For the film systems (no polystyrene included), only 1 uL pre-polymerization mixture was deposited within the O-ring. This gave, according to the formula for calculating the volume of a cylinder: $V=\pi r^2 h$ the layer thickness: $h= V/\pi r^2$ The ID of the O-ring used to fence the area for deposition of polystyrene and pre-polymerization mixture on the QCM chip was 7 mm, giving the r = 3.5 mm. → h of the polymer film = 0.025984 mm ≈ 26 µm

For the systems with PS beads, we have to account for the packing coefficient of spheres (the shape of the PS beads) in a cylinder (the volume fenced off with the O-ring). The packing coefficient of spheres in a cylinder is 0.74048. This gives that, when depositing 1 uL of pre-polymerization mixture on a drop coated area, this will fill the remaining 0.25952 volume, which is not packed by the PS beads. This gives that the height resulting from the PS drop coated systems is the height of the thin film systems/0.25952. Thus, 0.25952 -> 0.025984/0.25952 = 0.1001 mm ≈ 100 µm.

The ID of the O-ring used to fence the area for deposition of polystyrene and pre-polymerization mixture on the QCM chip was 7 mm. Herein 30 uL of polystyrene suspension in water was deposited. The liquid of the suspension was subsequently evaporated. Latex beads: 10% solids -> in 30 uL suspension, 3 uL PS beads. Thus 3 uL is the resulting evaporated volume. The height of the PS beads after evaporation of water, before accounting for the packing coefficient: $h= V/\pi r^2$ → h= 0.077853 mm. Then, the packing coefficient of cylinders is: 0.74048. Accounting for this gives the height: 0.077853/0.74048 = 0.10527 mm.

The height of the evaporated PS beads, if optimal packing is assumed, is slightly higher than the height that results of the pre-polymerization mixture if it fills the space between the PS beads. Thus, we may assume that there should be some ”uncovered” PS beads, which may come in contact with toluene so that they can be extracted out and form pores in the polymer. The height of the pre-polymerization mixture is thus what limits the height of the layer, i.e. ≈ 100 µm (after toluene wash).

3. QCM experiments

3.1 QCM experiments set-up

The analytical performance of the polymer covered QCM chips was investigated under flow injection analysis (FIA) conditions, by placing the chips in QCM holders (provided by Attana AB), using the Attana Cell 200 instrument (Attana AB). All QCM experiments were done at 20 °C, using a 1:1 mixture of methanol and a 0.1 M solution of sodium acetate in H_2O (pH 7) as the carrier solvent. This was infused by a peristaltic pump integrated in the QCM instrument with the flow rate 50 µL/min. Before doing any analyte injections, the frequency of the QCM chips in the carrier solvent was allowed to stabilize, as defined by a resonant frequency change of ≤0.5 Hz over 300 s. The analyte to be studied (150 µL) in a 1:1 mixture of methanol and a 0.1 M solution of sodium acetate in H_2O (pH 7) was injected with a 250 µL gas tight syringe (ID 10.9 mm, Kloehn) using the injection valve of the QCM instrument. At least three injections were done for each analyte on each polymer system and the average value is used for FIA calibration plots. The obtained data was collected with the software Attester (version 1.5.3, Attana AB), and analyzed with the software Attana Evaluation (version 3.3.4, Attana AB). The sensitivity was obtained from the slope of the calibration plots obtained by plotting the resonant frequency changes detected when injecting the respective analytes on the polymer films, as a function of the analyte concentration.

3.2 Analyte solutions for the QCM experiments

Samples for the QCM-FIA experiments were prepared by dissolving **L-TSA**, **D-TSA**, pyridoxamine dihydrochloride (**6**), phenylpyruvic acid (**7**), pyridoxal hydrochloride (**2**), L-phenylalanine (**L-11**) and D-phenylalanine (**D-11**), in a 1:1 mixture of methanol and a 0.1 M solution of sodium acetate in H_2O (pH 7), containing 1 mM, 5 mM, 10 mM, 20 mM, 30 mM, and 50 mM of each analyte. The structures of the compounds analyzed are shown in Scheme S1.

4. Scanning electron microscopy

The polymer films, deposited on silicon substrates (preparation of silicon substrates with polymer films is described in sections S2.2 and S2.3) were investigated with scanning electron microscopy (SEM) using a LEO Ultra 55 instrument (Carl Zeiss AG, Oberkochen, Germany) equipped with a field emission electron gun. Initially, polymer films were sputter coated with a thin layer of palladium

using an LEICA EM SCD 500 sputtering unit and placed on black carbon tape attached to alumina stubs before being inserted in the SEM instrument. A 3 kV potential was applied to the electron gun to generate the electron beam used to scan the polymer particles.

5. Fourier-transform infrared spectroscopy

FTIR analyses were performed using an Agilent Cary 630 FTIR Spectrometer (Agilent Technologies) equipped with KBr optics and complementary diamond attenuated total reflectance (ATR) sampling accessory. The polymer coated substrates were placed on a type IIa diamond crystal, and measured with ATR. This system is configured with the Agilent MicroLab FTIR Software (Agilent Technologies) for collecting a background spectrum and a sample spectrum. The samples were recorded within 400-4000 cm^{-1} range with 32 scans and 4 cm^{-1} resolutions.

6. References

1. J. Svenson, N. Zheng and I. A. Nicholls, *J. Am. Chem. Soc.*, 2004, **126**, 8554–8560.

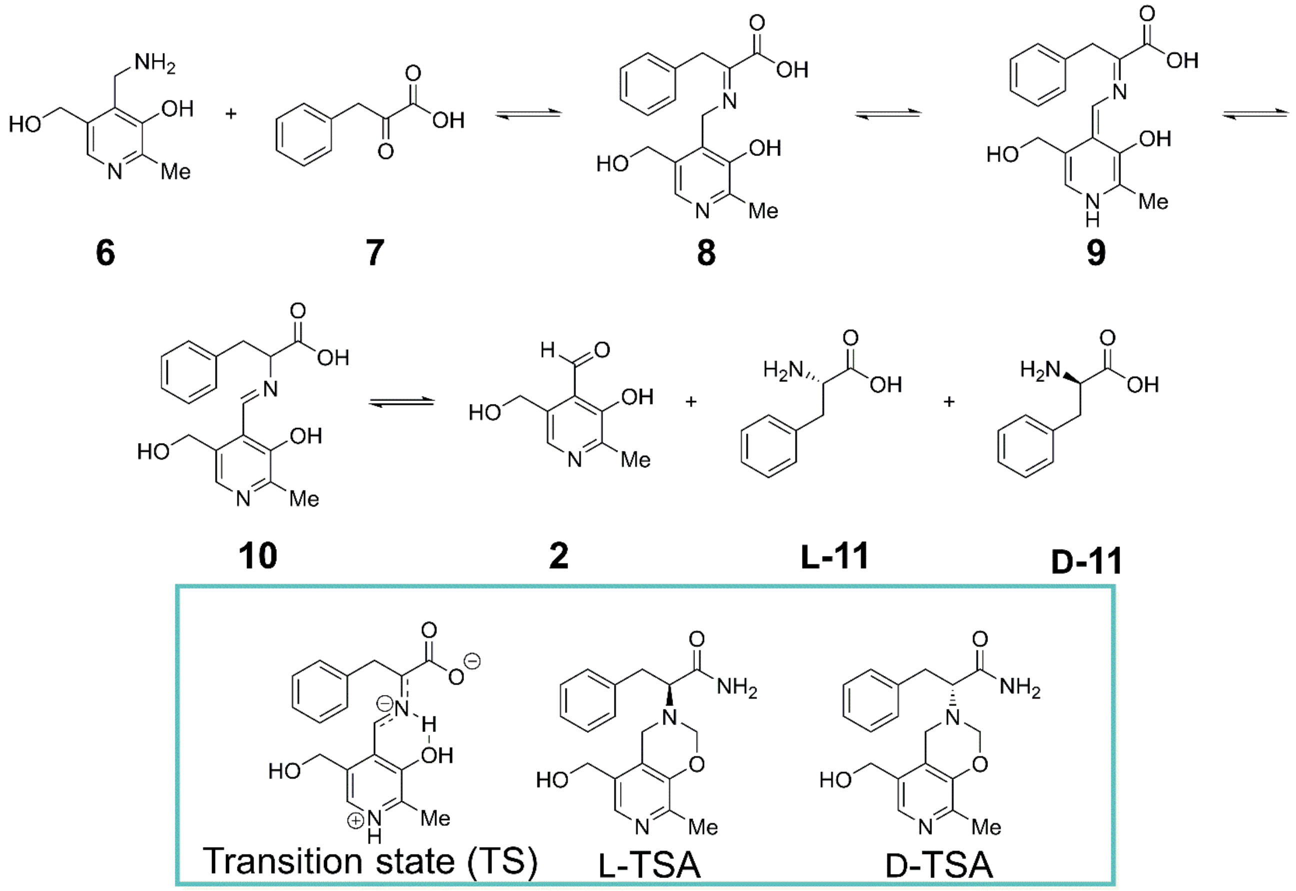

Scheme S1. The analytes relevant to the transaminase reaction. The compounds displayed in the scheme are: pyridoxamine (**6**), phenylpyruvic acid (**7**), pyridoxal (**2**), L-phenylalanine (**L-11**) and D-phenylalanine (**D-11**), **L-TSA** (**4**) and **D-TSA** (**5**).

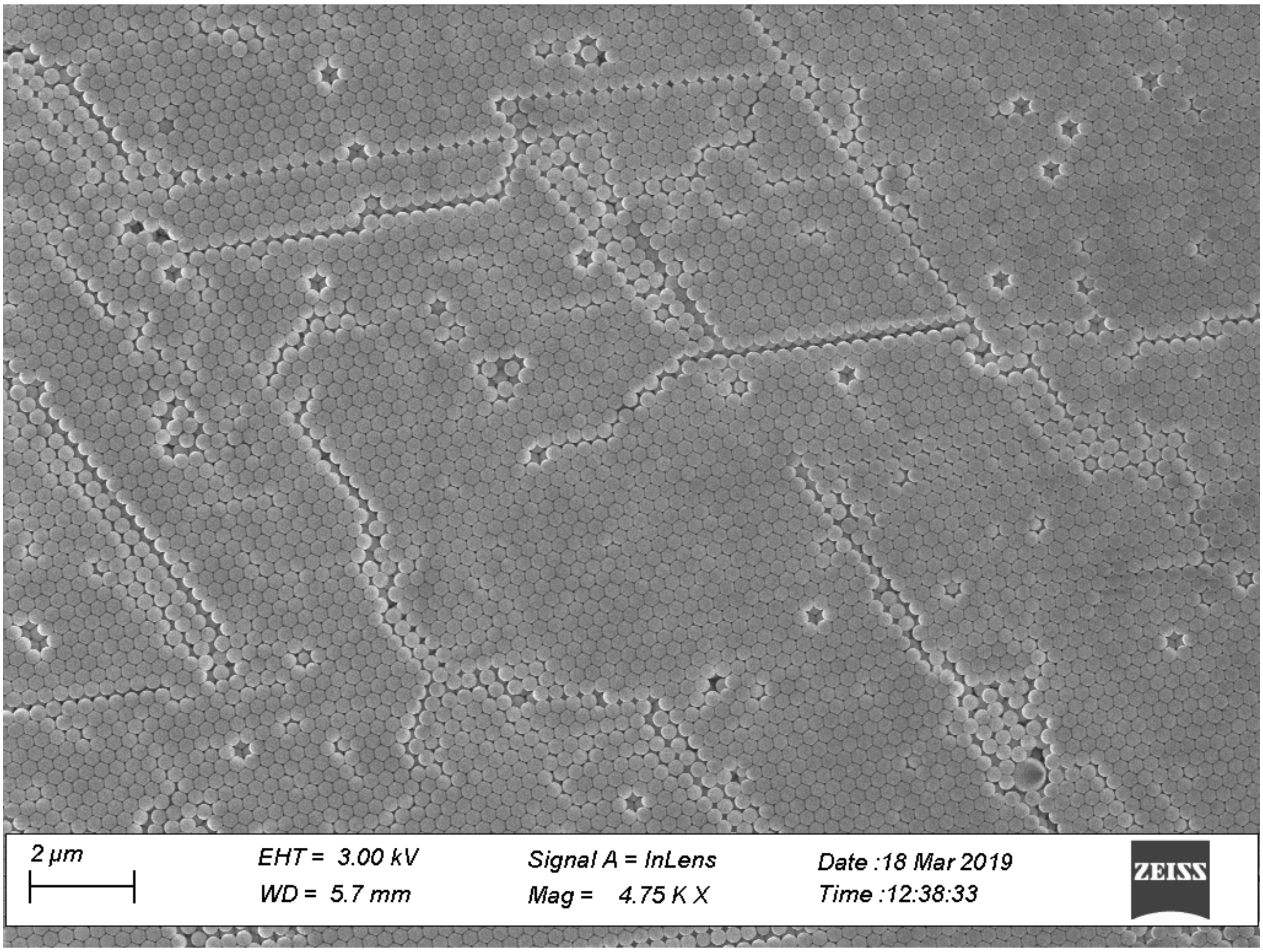

Figure S1. Scanning electron micrographs of 300 nm-diameter polystyrene sacrificial beads drop coated on SiO_2@Au-coated quartz crystal resonators.

(A)

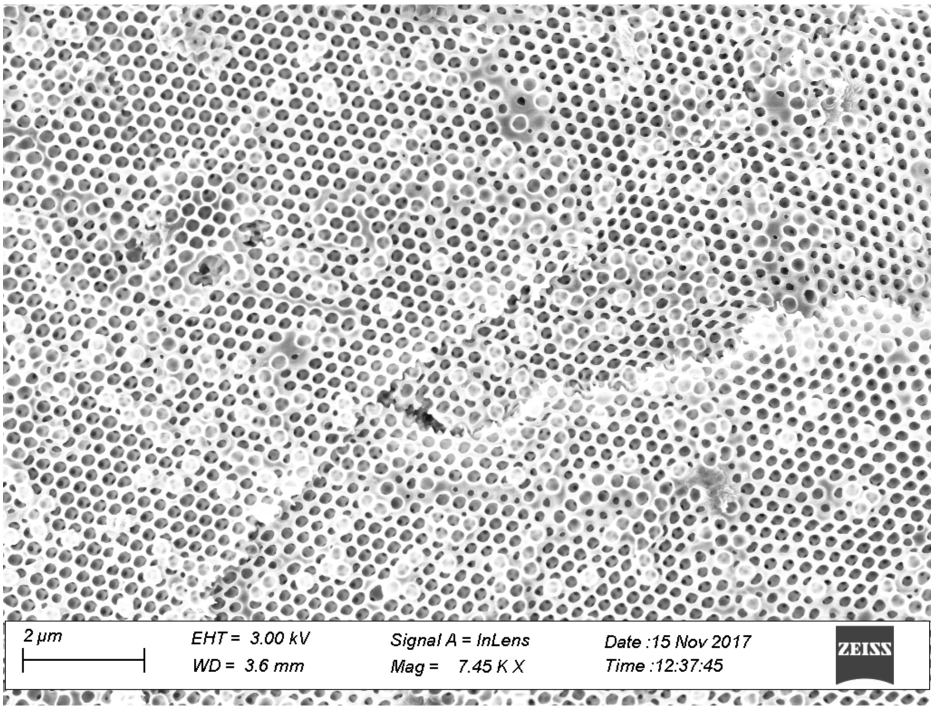

(B)

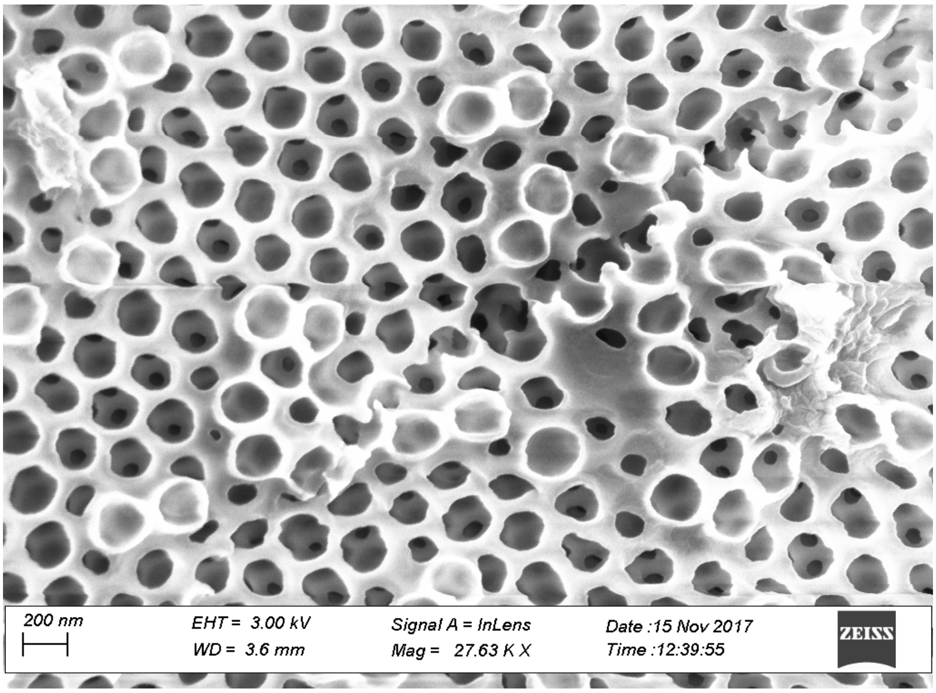

Figure S2. Scanning electron micrographs obtained for the polymer system imprinted with L-TSA, prepared in *n*-heptane (P4), drop-coated with two layers of 30 μL polystyrene suspension with A) lower and B), higher magnifications.

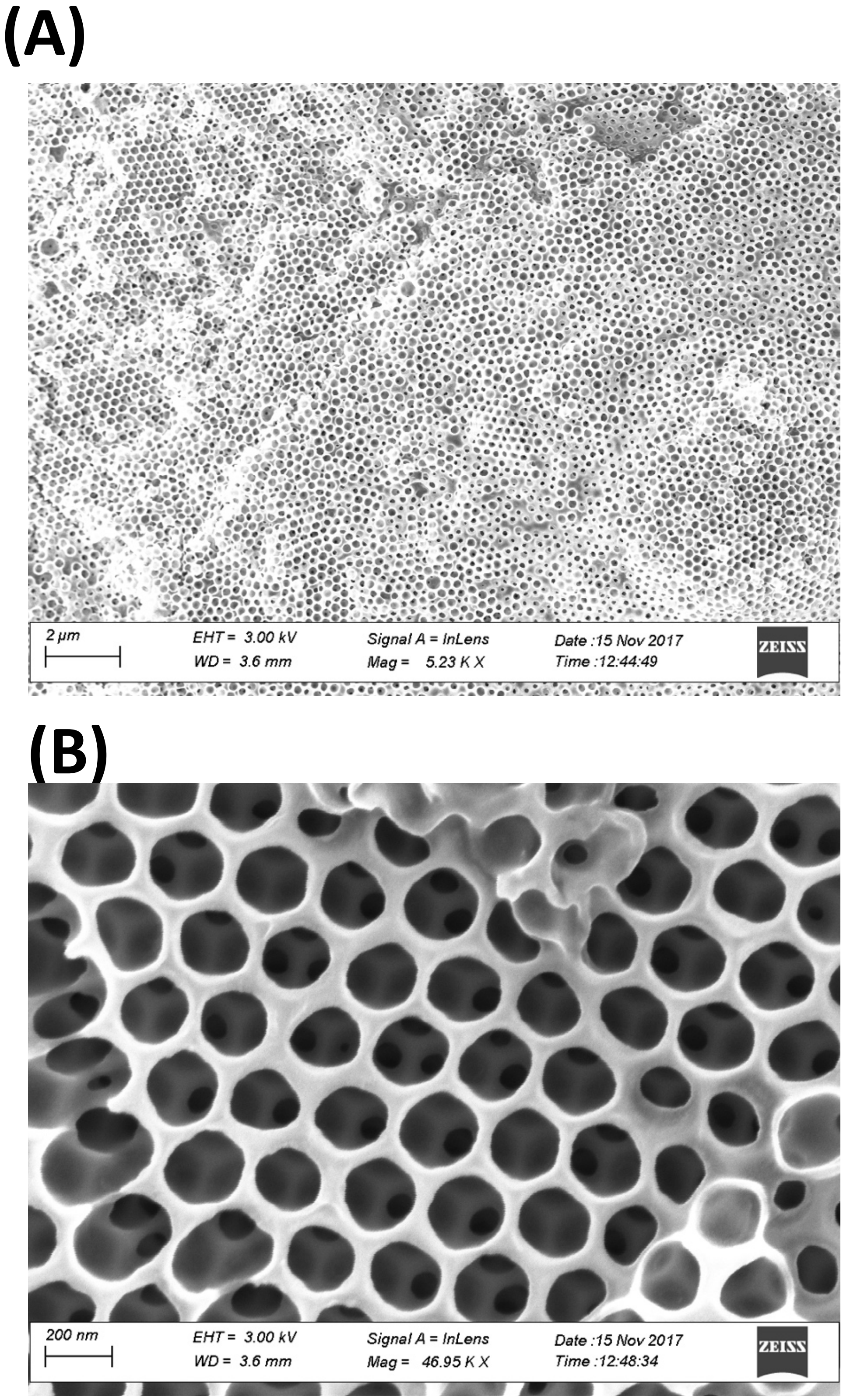

Figure S3. Scanning electron micrographs obtained for the polymer system imprinted with L-TSA (P6), prepared in the absence of any solvent, drop coated with two layers of 30 µL polystyrene suspension with A) lower and B), higher magnifications.

(A)

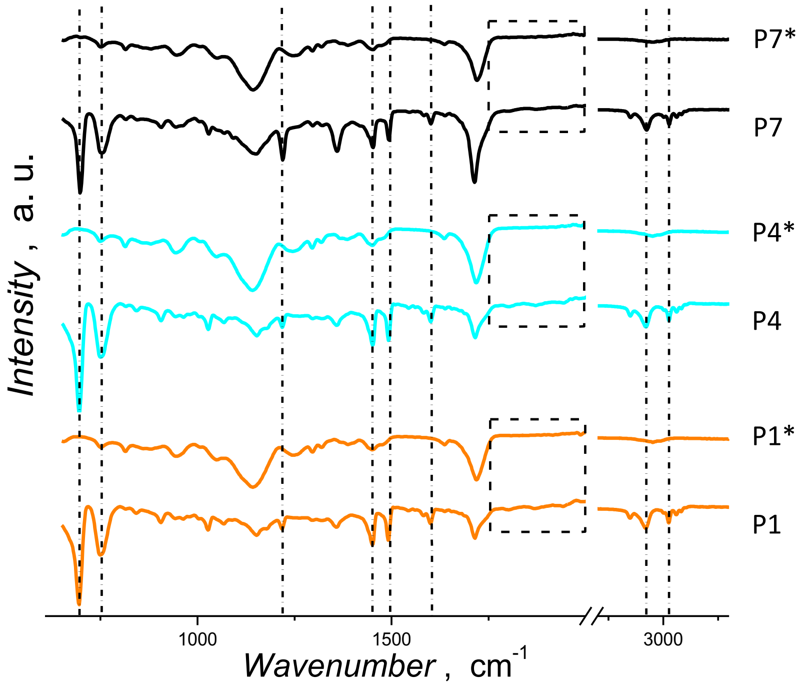

(B)

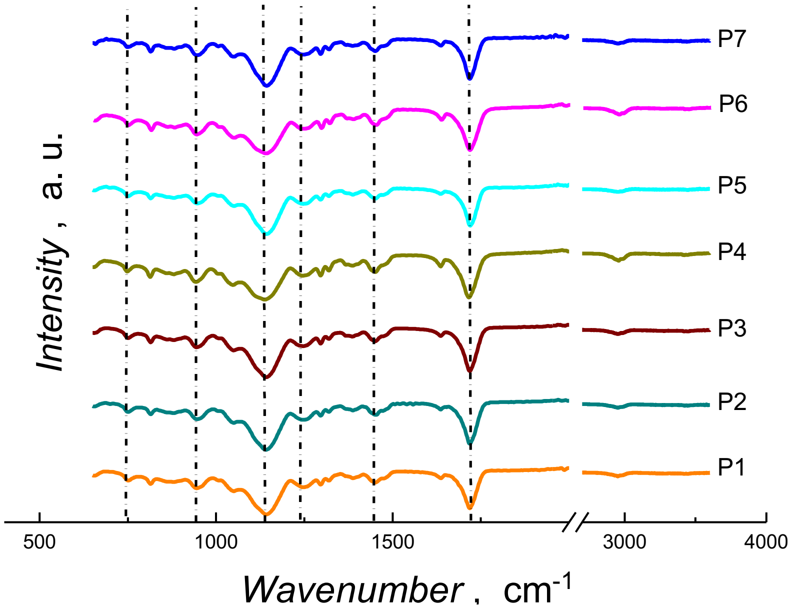

Figure S4. A) FTIR spectra of P1, P4 and P7 polymer systems before and after (*) washing in toluene to remove polystyrene beads, B) FTIR spectra of the polymer systems P1-P7 after wash with toluene, ethanol and a 5 mM solution of NaOH in H_2O. The bands marked are in (A) ρC-H 696, ρC-H 752, δ(C-H) 1216, νC=C 1449, νC=C 1492, νC=C 1602, νC-H 2922 and νC-H 3025 cm^{-1}, as well as overtones in the box around 1800-2000 cm^{-1} and in (B) ρC-H (750 and 900 cm^{-1}), νC-O (1145 and 1250 cm^{-1}), δ(C-H) (1455 cm^{-1}), νC=O (1710 cm^{-1}), arising from respective vibrational modes.

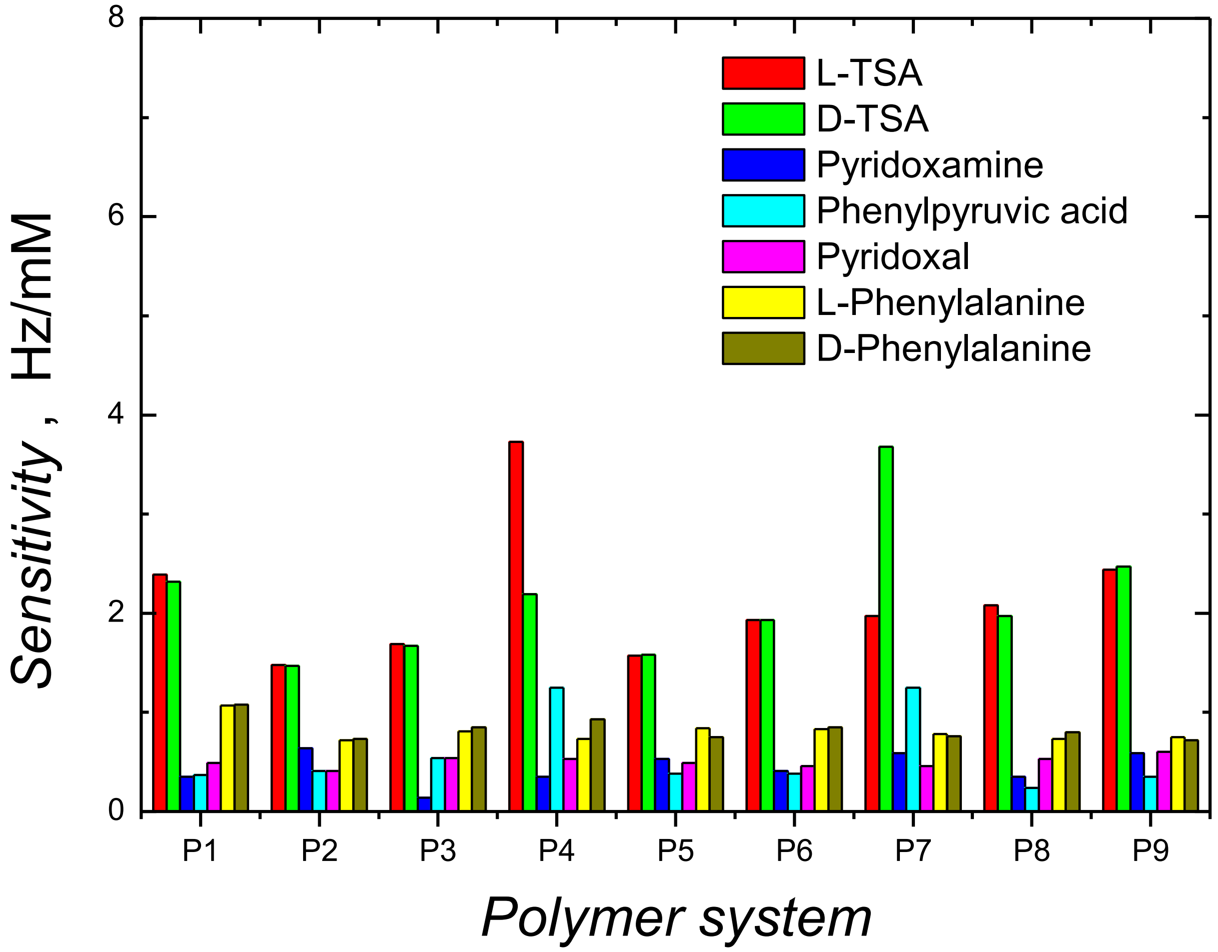

Figure S5. Histogram of sensitivity of polymer systems (Table 1, Table S1 and Table S2) obtained from slope of the FIA calibration plots of various analytes on different polymer systems

Table S1. Composition of the prepared polymer systems.

Polymer system	Molecular template	Sacrificial template	Functional monomer	Crosslinker	Initiator	Solvent
P1	-	PS beads	MAA (47.6 µmol)	EGDMA (0.221 mmol)	AIBN (7.92 µmol)	*n*-Heptane
P2	-	-				*n*-Heptane
P3	-	PS beads				-
P4	3.97 µmol L-TSA	PS beads				*n*-Heptane
P5	3.97 µmol L-TSA	-				*n*-Heptane
P6	3.97 µmol L-TSA	PS beads				-
P7	3.97 µmol D-TSA	PS beads				*n*-Heptane
P8	-	-				-
P9	3.97 µmol L-TSA	-				-

Table S2. The sensitivity of the studied polymer systems (obtained from the slope of the calibration plots) for all investigated analytes. Value in parenthesis is the correlation co-efficient of the sensitivity.

**Analyte**	**Sensitivity (Hz/mM)**								
	**P1**	**P2**	**P3**	**P4**	**P5**	**P6**	**P7**	**P8**	**P9**
**L-TSA**	2.39 (0.9899)	1.48 (0.9971)	1.69 (0.9962)	3.73 (0.9978)	1.57 (0.9991)	1.93 (0.9949)	1.97 (0.9971)	2.08 (0.9986)	2.44 (0.9993)
**D-TSA**	2.32 (0.9958)	1.47 (0.9897)	1.67 (0.9984)	2.19 (0.9957)	1.58 (0.9972)	1.93 (0.9989)	3.68 (0.9984)	1.97 (0.9982)	2.47 (0.9986)
**Pyridoxamine**	0.35 (0.9758)	0.64 (0.9919)	0.14 (0.9314)	0.64 (0.9940)	0.53 (0.9799)	0.41 (0.9803)	0.59 (0.9964)	0.35 (0.9680)	0.59 (0.8090)
**Phenylpyruvic acid**	0.37 (0.9946)	0.41 (0.9903)	0.54 (0.9758)	1.25 (0.9897)	0.38 (0.9892)	0.38 (0.9721)	1.25 (0.9931)	0.24 (0.9636)	0.35 (0.9822)
**Pyridoxal**	0.49 (0.9980)	0.41 (0.9919)	0.54 (0.9972)	0.47 (0.9900)	0.49 (0.9859)	0.46 (0.9959)	0.46 (0.9889)	0.53 (0.9783)	0.60 (0.9896)
**L-phenylalanine**	1.07 (0.9932)	0.72 (0.9886)	0.81 (0.9967)	0.83 (0.9992)	0.84 (0.9994)	0.83 (0.9773)	0.78 (0.9929)	0.73 (0.9761)	0.75 (0.9902)
**D-phenylalanine**	1.08 (0.9825)	0.73 (0.9451)	0.85 (0.9919)	0.93 (0.9993)	0.75 (0.9984)	0.85 (0.9904)	0.76 (0.9991)	0.80 (0.9986)	0.72 (0.9951)
